# Ewing sarcoma of the oral cavity. A review

**DOI:** 10.4317/jced.53575

**Published:** 2017-02-01

**Authors:** Maria Margaix-Muñoz, José Bagán, Rafael Poveda-Roda

**Affiliations:** 1 DDS, PhD. Associate Professor of Oral Medicine. Department of Stomatology, University of Valencia. Valencia (Spain); 2 MD, DDS, PhD. Charmain of Oral Medicine. Department of Stomatology, University of Valencia. Head of Stomatology and Maxillofacial Surgery Service. General Universitary Hospital of Valencia. Valencia (Spain); 3 MD, DDS, PhD. Staff physician. Stomatology and Maxillofacial Surgery Service. General Universitary Hospital of Valencia. Valencia (Spain)

## Abstract

**Objectives:**

A review is made of the clinical, diagnostic, therapeutic and survival characteristics of Ewing sarcoma (ES) of the oral cavity.

**Material and Methods:**

A systematic literature search was carried out, with restrictions referred to time (1960-2014), language (English and Spanish) and type of study (case reports, letters, datasets, reviews). The following MeSH terms and boolean operators were used: Ewing AND Sarcoma AND [tongue, jaw, maxilla, cheek, condyle OR temporomandibular, floor AND mouth, gum OR gingiva, palate OR palatal, lip, uvula, head AND neck].

**Results:**

Seventy-one cases of ES of the oral cavity were documented from 53 articles. The main differences versus ES of other locations were a younger age at manifestation, a shorter time from symptoms onset to diagnosis, and swelling as the most frequent clinical manifestation versus swelling and pain in the rest of disease locations. The way in which ES manifests in the oral cavity is varied and comprises dental displacement (19.7%), dental mobility (7%), root reabsorption (5.6%), destruction of the dental follicle (4.2%), premature exfoliation (4.2%) and paresthesia of the chin (2.8%). Metastatic neck adenopathies appear in 11.3% of the cases. Significant differences in survival are observed between patients with a complete diagnosis of ES (hematoxylin-eosin staining, PAS positivity, CD99 positivity) and those with an incomplete diagnosis.

**Conclusions:**

Ewing sarcoma of the oral cavity presents a series of specific features that distinguish it from ES of other locations.

** Key words:**Primitive neuroectodermal tumor, PNET, Ewing sarcoma, Ewing tumor, sarcoma, oral cavity.

## Introduction

Ewing sarcoma (ES) was first described in 1921 by James Ewing, who referred to the disease as diffuse endothelioma of bone, because he assumed that the tumor originated from the vascular component of bone. Since then the disease has received a number of names: perithelioma, endothelial myeloma, reticuloendothelioma, reticular sarcoma and intramedullary sarcoma - thus evidencing the uncertainty regarding the true origin of the tumor. In 1928, Oberling proposed the term Ewing sarcoma, thereby obviating the debate about the causal cells. The designation proposed by the World Health Organization (WHO) is Ewing sarcoma/primitive neuroectodermal tumor (ES/PNET), based on the assumption that both entities correspond to the same process, since they share the same genetic alteration (translocation 11:22) in over 95% of the cases (1).

The origin of ES remains unclear, though the tumor is suspected to derive from the neuroectodermal cells. The immature reticular cells and primitive mesenchymal cells of the bone marrow (2) have also been proposed as possible origins of the tumor.

Ewing sarcoma is the fourth most common bone malignancy after myeloma, osteosarcoma and chondrosarcoma (though some authors rank it ahead of chondrosarcoma) (3), and it is the second most frequent malignant bone tumor in infancy and childhood, after osteosarcoma (4) - with an estimated incidence of 2.93 cases/million inhabitants under 20 years of age/year (5). Ewing sarcoma represents 1% of all malignant tumors in children (6) and between 4-10% of all bone malignancies (7). Its behavior is that of an aggressive malignant tumor, and it has been estimated that metastases are already present at the time of diagnosis in 15-28% of the cases (6,8). However, when occult micrometastases are considered, some publications raise this percentage to as high as 80% (9). The tumor shows a predilection for the male gender in 2.1-2.4:1 proportion (5,9), and it is 10 times more frequent in Caucasian children than in black children (10).

Hematoxylin-eosin staining (HE) defines ES as belonging to the group of small blue round cell tumors - a series of very aggressive tumors with a high incidence in children and adolescents. The periodic acid-Schiff technique (PAS), which stains carbohydrates red, is usually positive in ES, revealing the presence of intracellular glycogen granules, which help differentiate the disease from tumors of neural lineage. Ewing sarcoma is also positive for cluster of differentiation 99 (CD99), a protein encoded for by the myc2 gene located on the short arm of chromosomes X and Y, and which is highly sensitive for small blue round cell tumors in children, particularly ES/PNET, lymphoblastic lymphoma and lymphoblastic leukemia.

The most precise diagnostic factor is translocation t(11;22)(q24;q12), which is detected in 85-90% of all cases of ES (9). This translocation gives rise to a fusion gene called EWS/FLI1 (Ewing Sarcoma/Friend leukemia integration 1 transcription factor). In approximately 5% of all cases, the EWS gene is implicated in other types of translocations, namely t(11,22)(q12;q12) and t(7;22)(p22;q12), which give rise to the fusion genes EWS-ERG and EWS-ETV1, respectively (11). One-fourth of the tumors present p16 and p53 alterations, which is associated to more aggressive behavior of the disease and a poorer response to chemotherapy (12).

The most frequent location of ES is in the long bones (58%), pelvis (20%) and ribs (7%) (7). Ewing sarcoma of the head and neck is less common, representing about 3% of all cases (13,14). It accounts for 10.5% of all primary malignancies of the mandible (11). Ewing sarcoma of the oral cavity has a series of particularities which are addressed in the present study.

The tumor is still associated to a poor prognosis, though the latter has improved considerably as a result of the introduction of recent treatment protocols warranted by clinical trials. Treatment initially consisted of radiotherapy and surgery, and was associated to a very poor survival rate of about 20% after three years (15) and of 5-8% after 5 years (10). The studies of Rosen *et al.* (16), administering neoadjuvant chemotherapy, radically changed the prognosis of the disease. Current management is based on neoadjuvant chemotherapy, surgery / radiotherapy, and adjuvant chemotherapy. With this strategy, overall survival currently varies between 59% and 81% after three years, according to whether adults or children are involved, and the disease-free survival rate is about 60% after 5 years (17,18). This percentage decreases to 30% after 3-5 years in the case of disseminated disease at the time of diagnosis (19,20).

The present study analyzes the available clinical information of ES of the oral cavity.

## Material and Methods

A structured Medline search was made with the following restrictions and search sequences:

Time restriction: 1 January 1960 to 30 June 2014.

Language restriction: English and Spanish.

Publication restriction: human, case reports, letters, datasets, reviews.

The following MeSH terms and boolean operators were used:

Ewing AND Sarcoma AND [tongue, jaw, maxilla, cheek, condyle OR temporomandibular, floor AND mouth, gum OR gingiva, palate OR palatal, lip, uvula, head AND neck].

A total of 297 literature references were identified. After manually filtering the articles found to be duplicated in the different search strategies and those that lacked clinical information, a total of 53 publications were finally selected. Of these, 45 corresponded to a case description, while two, three, two and one article reported 2, 3, 4 and 5 clinical cases, respectively. A total of 71 clinical cases of ES of the oral cavity were compiled. The following information was collected for each case, where available: patient gender, age, tumor location, time to first consultation, personal history, initial diagnostic impression, first manifestation of the disease, other subsequent clinical manifestations, imaging findings (conventional X-rays, panoramic X-rays, computed tomography [CT], magnetic resonance imaging [MRI], etc.), metastases and the technique used to diagnose metastatic spread, treatment provided, and survival.

A descriptive analysis was made, with frequency distributions for qualitative variables and calculation of the mean and standard deviation (SD) (with the corresponding confidence interval) in the case of quantitative variables. Survival curve was plotted using the Kaplan-Meyer method. Lastly, the data of the present study were contrasted with the historical information referred to ES of systemic and craniofacial location.

## Results

The documented cases were published in 53 articles in 29 different journals. The great majority of the publications (45/71; 63.4%) corresponded to single clinical cases. The journal with the largest number of published cases was Oral Surg Oral Med Oral Pathol Oral Radiol (11 cases), followed by J Craniofac Surg (8 cases) and Otolaryngol Head and Neck Surg (7 cases).

A little over one-half of the cases were published in buccodental journals (56.3%), while the rest were published in general medical journals or journals corresponding to other specialties (Oncology, Otorhinolaryngology, Radiology, etc.).

Although the study design contemplated data compilation starting in the year 1960, most of the cases were of recent publication. Over one half (56.3%) were published in the period 2000-2014, while less than one-fifth were published between 1960-1990 (19.7%).

Ewing sarcoma of the oral cavity showed a slight female predominance (50.7%), with a mean patient age of 15 years and 3 months ± 8 years and 7 months (SD) (range: 1-43 years). The tumor was fundamentally located in the mandible (69% of the cases). The first clinical manifestation was swelling (46.5%), followed by swelling and pain (12.7%). One out of every 5 cases (21.1%) was initially mistaken for dental infection. In descending order of frequency, the disease clinically manifested in the form of swelling (70.4%), pain (28.2%), dental displacement (19.7%), neck adenopathies (11.3%), fever (9.9%), dental mobility (7%), root reabsorption (5.6%), destruction of the dental follicle and premature dental exfoliation (4.2%). Ewing sarcoma was predominantly of hard consistency (29.6%), and the overlying mucosa was usually of normal appearance (15.5%). The imaging techniques characteristically revealed a radiotransparent lesion (38%) with poorly defined limits (40.8%).  Table 1 describes the most relevant clinical data compiled in our study.

**Table 1:**
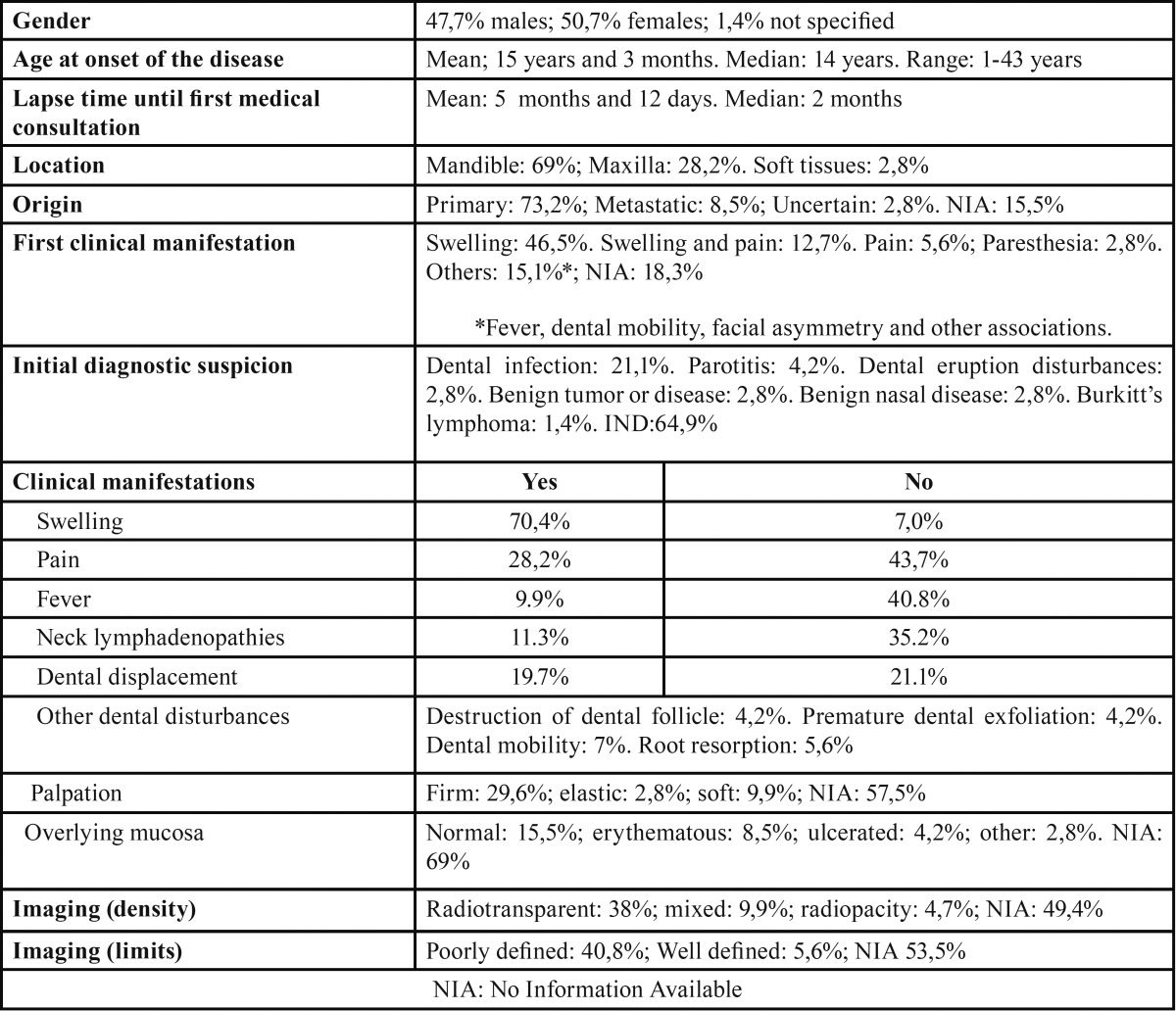
Manifestations of Ewing Sarcoma of the oral cavity.

**Figure 1:**
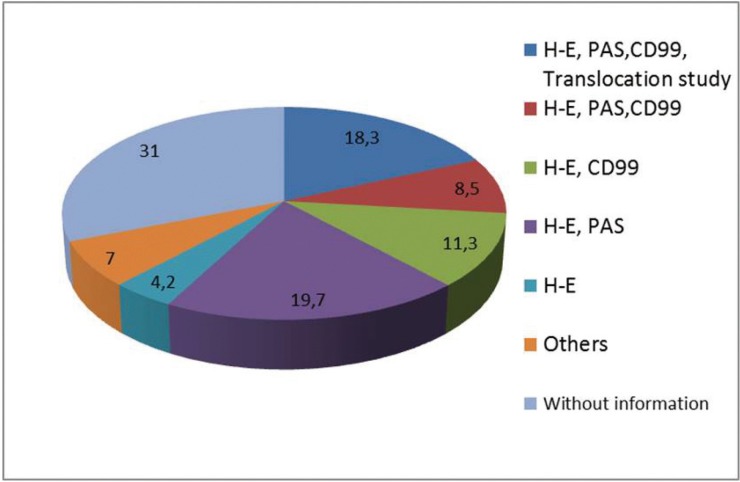
Histological criteria employed for diagnosis of Ewing Sarcoma on clinical cases analyzed (H-E: hematoxylin-eosin; CD99: Cluster of Differentiation 99; PAS: Periodic acid-Schiff technique. Translocation study: translocation study 11,22).

In 31% of the cases no description was provided about the diagnostic methods used. In 18.3% of the patients the diagnosis was established from HE and PAS staining, CD99 and the translocation 11:22 study findings. In 8.5% of the cases HE and PAS staining and CD99 were used, while in 11.3% of the patients only HE and PAS staining were employed. The histopathological criteria leading to the diagnosis of ES of the oral cavity in each case are summarized in figure 1.

**Figure 2:**
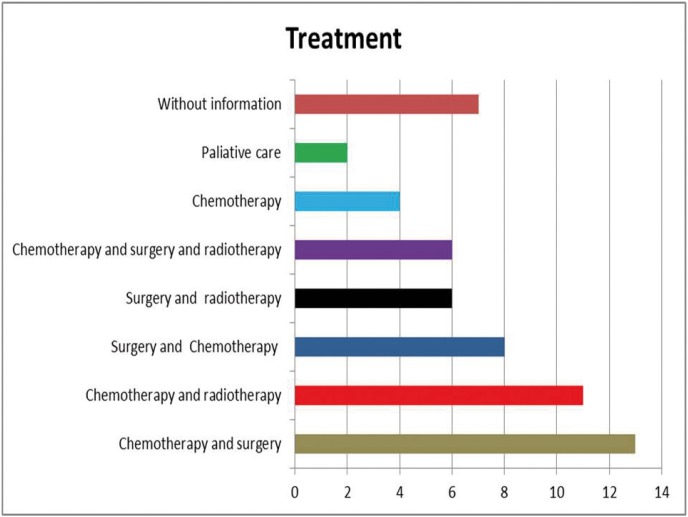
Management strategies described in Ewing Sarcoma of the oral cavity.

The most common management strategy was chemotherapy and surgery, followed by chemotherapy and radiotherapy. The different treatment modalities are described in figure 2.

**Figure 3:**
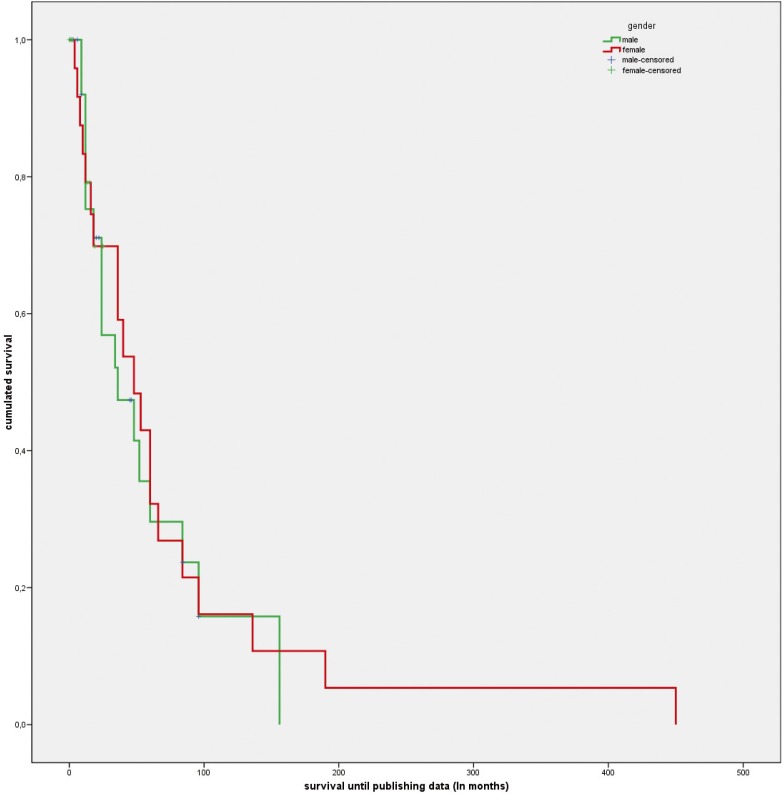
Survival analysis based on Kaplan-Meyer method in Ewing Sarcoma of the oral cavity. Comparison between genders.

 Table 2 and figures 3a and 3b show the survival data of the patients with ES of the oral cavity, with the corresponding gender comparison.

**Table 2:**
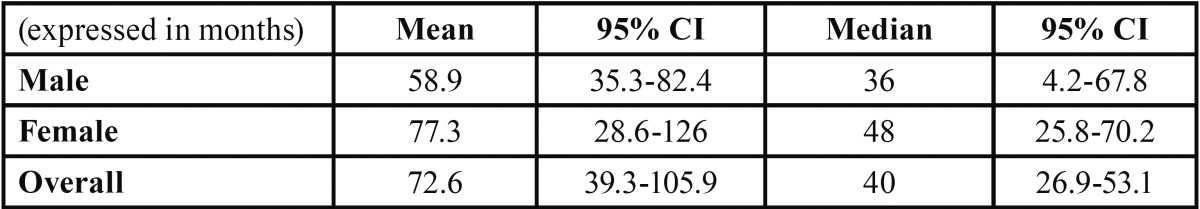
Survival of Ewing sarcoma. Mean and median with confidence interval (95% CI).

The size of the tumor at the time of diagnosis was reported in 26 cases (36.7%). Seventeen of them mentioned the clinical size of the lesion, while 6, 1 and 2 assessed lesion size from the CT scan, MRI study and measurement of the surgical piece, respectively. The most common tumor size was 3-5 cm in diameter (in 15 of the 26 cases for which information was available; 57.7%), with a range of 1-12 cm.

Of the total 71 cases, 6 (8.5%) corresponded to oral metastatic spread of a primary tumor in some other location, and in one case (published in 1968 (21)) the primary or metastatic nature of the lesion could not be established from the description of the clinical case. The remaining 64 tumors corresponded to primary malignancies of the oral cavity. Most of the publications either failed to specify whether metastatic disease was present or simply stated that there was no metastatic spread, without explaining how this was confirmed (33/64; 51.6%). In those cases where such information was provided, metastatic X-ray series, CT and scintigraphy (with or without bone marrow biopsy) were the most commonly used techniques ( Table 3).

**Table 3:**
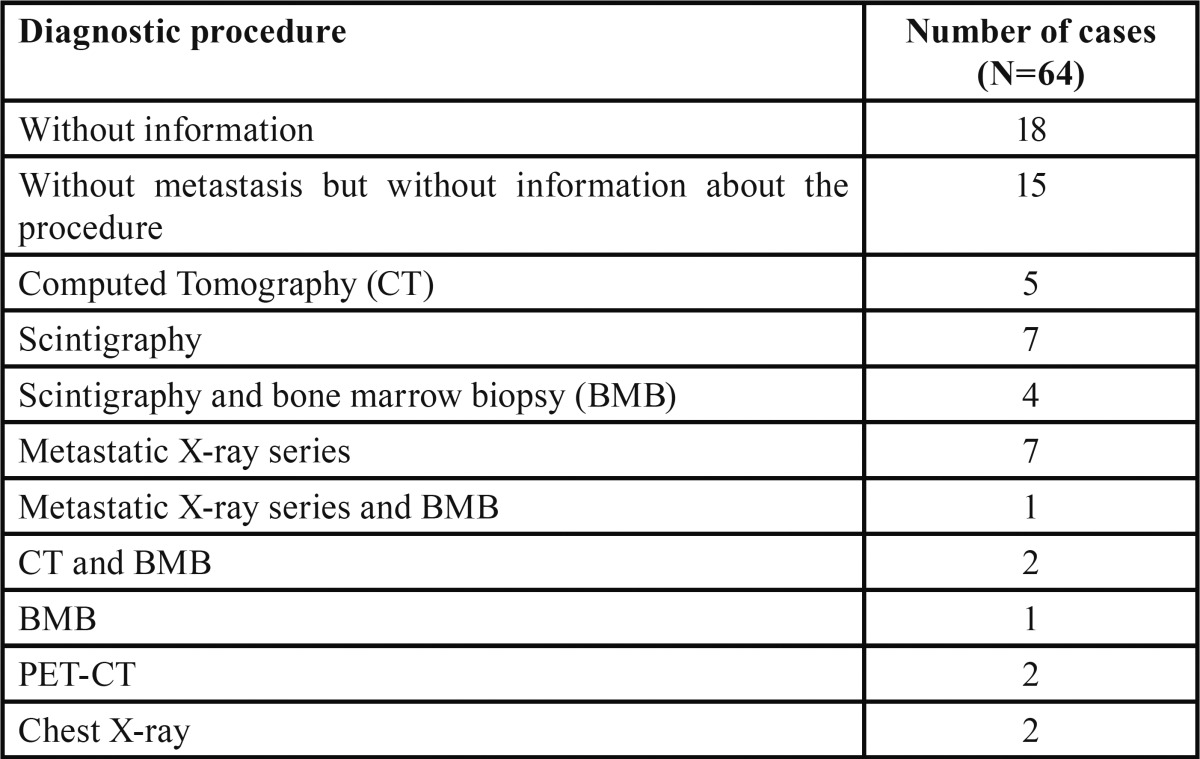
Diagnostic procedures for metastasis detection in Ewing Sarcoma.

## Discussion

Overall, Ewing sarcoma is more prevalent in males than in females when considering all tumor locations. Some publications specifically referred to the head and neck report a male predominance of between 1.4:1 (22) and 2.4:1 (5). This gender difference is not seen in ES of the oral cavity and appendages, where the gender distribution appears to be balanced, with only a very slight female predominance of 1:1.05 ( Table 1). Some authors comment that the gender difference is noted above the age of 13 years (23). However, the data obtained in our study indicate otherwise, with a slightly greater predominance in females among patients over 13 years of age (1:1.25).

Patient age at onset of the disease (mean: 15 years and 3 months ± 8 years and 7 months; median: 14 years) was lower than reported for ES of any location (18 years) (17). With regard to ES of the oral cavity, our data coincide with those reported by other series (11,24), but differ clearly from the findings of Gupta *et al.* (10), who reported a mean patient age for head and neck locations of 10.9 years. The published data correspond to case series involving very few patients, and this could explain the broad variability observed. Our data also confirm the accumulation of most cases (83.1%) in the 5-25 years age range, as already reported for ES of any location (6).

The time from first clinical manifestation of the disease to first medical consultation was reported in almost two-thirds of the cases (63.3%), and ranged widely (mean: 5 months and 12 days ± 8 months; range: 1-48 months). We therefore consider reporting of the median to be clinically more useful (2 months). This delay in first consultation is slightly shorter than in other malignancies of the oral cavity (25), and this may be related to the rapid and aggressive growth of the tumor, and the fact that it commonly manifest in children, which are usually monitored more closely. The mentioned delay is also slightly shorter than that reported for ES of other locations (26), probably because of the greater visibility and accessibility of tumors of the oral cavity. A relationship has been described between tumor location in the pelvis, legs and arms and a longer time in establishing the diagnosis (27).

The mandible was the most frequent location of the tumor in both our study limited to the oral cavity and in studies involving the head and neck - representing over two-thirds of all the lesions (68%). Our observation coincides with that of Gupta *et al.* (10), who in a series of 65 cases of primary sarcoma of the jaws recorded a mandibular location in 70.7% of the cases, with a predilection for the ascending ramus (28,29). It has been suggested that the larger amount of bone marrow present in the ascending ramus would account for the greater prevalence of these tumors in that location (30).

Despite the strong tendency of these tumors to produce distant metastases, only 8.5% of the documented cases were oral metastases of primary tumors in other locations. This is consistent with the observations of other publications that have found approximately 90% of the mandibular malignancies to be primary tumors (28,31). In most of the metastatic presentations, the primary tumor was located in the ribs (66.6%).

It is accepted that the most frequent initial manifestation of the disease is in the form of swelling and pain, though in our study specifically centered on ES of the oral cavity the most frequent initial manifestation was swelling only. This was the case in one-half of the patients (46.5%), while the combination of swelling and pain as first manifestation of the disease was only recorded in a little over one of every 10 cases (12.7%). The oral cavity is easily accessible to exploration, and the bone structures do not lie under thick muscle layers as in other more common locations of ES such as the femur, pelvis or even the ribs. This may explain why tumors in these locations are identified later than tumors of the oral cavity, and consequently why pain is more often reported as first manifestation, whether associated to swelling or not (26).

Ewing sarcoma of the oral cavity has relevant features not found in other locations, and which are relevant for the dental surgeon, such as initial manifestation of the disease with mental nerve paresthesia, dental displacement, or dental mobility ( Table 1).

Information referred to the initially suspected diagnosis was only available in one of every three cases (35.1%), though it is interesting to note that the tumor was initially managed as a dental infection in 21.1% of the patients. It may be postulated that such confusion would be found to have occurred in over one-half of the patients if the required information were available in all cases. If the initial manifestation of the disease is easily confused with dental infection, evaluation must be made of those differentiating features capable of orienting the diagnosis towards malignancy, such as chin paresthesia, the absence of suppuration, the absence of dental lesions justifying an infectious process, the consistency of the lesion, possible general patient alterations, etc. - since diagnostic and management delays in such aggressive tumors can have fatal consequences. Other incorrect initial diagnostic impressions were altered dental eruption, parotitis or benign tumors ( Table 1).

In addition to being the most frequent initial manifestation of the disease, swelling was much more prevalent than the second most common manifestation, i.e., pain (70.4% versus 28.2%, respectively). An earlier diagnosis, and therefore sooner treatment, would contribute to avoid the appearance of pain.

The studied publications hardly mentioned the presence of metastatic neck adenopathies, which we documented in one out of every 10 cases (11.3%).

Ewing sarcoma of the oral cavity was more frequently of hard consistency than of elastic or soft consistency (29.6% versus 12.7%, respectively). This coincides with the observations of Mamede *et al.*, who described the tumor as initially being of hard consistency, followed by changes in consistency as the lesion gradually destroys the cortical layer (32).

Certain clinical features were not as frequent as those commented above, but are of special relevance to dental surgeons, since these are the professionals most likely to be consulted by patients with such manifestations: dental displacement, premature dental exfoliation or loss, non-physiological dental mobility, root reabsorption and destruction of the dental follicle. These manifestations were recorded in almost one-half of the patients with ES of the oral cavity (40.1%). Dental displacement was the most frequent alteration, being seen in at least one of every 5 cases (19.7%). In the presence of this clinical sign, the possibility of a malignant tumor should be considered, and if the patient is young (5-25 years of age), osteosarcoma and Ewing sarcoma must be included in the differential diagnosis.

In most cases the overlying oral mucosa was of normal appearance, since ES is a mesenchymal tumor that is diagnosed relatively early in oral locations - though in at least one of every 8 patients (12.7%) the mucosa presented erythema or ulceration.

Pathological fracture, which is relatively frequent in other locations (with an incidence of 15% when the tumor is located in long bones and up to 30% when specifically located at femoral level) (33), is rare in maxillary or mandibular tumors (23). In fact, we documented no such fractures in the 71 cases compiled in our study.

The radiological features of ES of the oral cavity correspond to a very aggressive, non-osteogenic malignancy. The tumor is pre-dominantly radiotransparent, with poorly defined limits and no peripheral sclerotic reaction. Some sporadic cases have described a radiopaque or mixed pattern, with a well defined contour. The sunray or onion peel pattern characteristic of malignant bone tumors is very infrequent in ES of the oral cavity (34).

Histologically, ES of the oral cavity is currently regarded as a tumor of neuroectodermal origin. Hematoxylin-eosin (HE) staining reveals an intense blue color that defines ES as belonging to the group of small blue round cell tumors, along with osteosarcoma, neuroblastoma, mesenchymal chondrosarcoma, lymphoma, metastatic small-cell lung cancer, rhabdomyosarcoma, synovial sarcoma, small round cell desmoplastic tumor, eosinophilic granuloma and malignant melanoma (35,36).

In turn, the PAS stain (specific of intracytoplasmic glycogen granules) is positive in over 90% of these tumors (37) and is useful in differentiating ES from tumors of neural lineage, which do not show PAS positivity.

Immunohistochemical techniques detect cluster of differentiation 99 (CD99) positivity in up to 98% of these tumors (38). Such techniques identify the products derived from translocation 11:22, but this is also observed in lymphoblastic leukemia-lymphoma, Hodgkin and non-Hodgkin lymphoma, and alveolar and embryonic rhabdomyosarcoma (35).

The tumor presents a genetic translocation between genes 11 and 22 (t;11:22) that gives rise to a fusion gene (Ewing sarcoma/Friend leukemia integration 1 transcription factor [EWS/FLI1]) with potent oncogenic action. This gene encodes for a protein that is currently considered to be the main factor responsible for the tumor.

Based on the study of Rosen in 1974 (17), the general management strategy for ES comprises initial chemotherapy followed by surgery and, subsequently, radiotherapy or chemotherapy depending on the initial treatment outcome. In the case of non-resectable tumors, treatment consists of chemotherapy, radiotherapy and subsequent chemotherapy.

As can be seen in Figure 2, this was the management strategy used in most patients. However, the above treatment scheme was not employed in a considerable number of cases, either because they predated the Rosen management protocol or because of limitations in the treatment possibilities (non-accessible tumors, patient refusal to receive or finance the treatment, etc.). In this regard, Bamamuth *et al.* reported that 45% of the patients with ES of any location did not receive adequate treatment (39).

Information referred to survival was available in 79.9% of the cases. The median survival was 40 months, with an apparent difference in median survival between males and females (36 months versus 48 months). However, the Kaplan-Meier analysis revealed no significant gender differences, due to the existence of important variance and wide confidence intervals (Fig. 3).

According to some publications (40), the diagnosis of ES requires at least two neuronal markers and evidence of translocation 11:22. In order to examine the possibility that some tumors may have been wrongly classified as ES, a survival analysis was performed of the 27 patients that at least presented HE and PAS positivity and CD99-positivity, comparing them with the rest of the cases based on the Kaplan-Meier method. An evident and statistically significant difference in survival was observed between the two groups (median 24 months; 95%CI 10.3-37.6 versus 60 months; 95%CI 40.5-79.5 months; chi-squared, log-rank test = 8.3; *p*=0.004), suggesting that some cases documented in the literature as ES probably corresponded to other types of tumors.

The published survival data on ES vary considerably, though it is assumed that tumors located in the head and neck region have a better prognosis than ES in other locations (80% versus 56% after three years) (16).
